# Diabetes Mellitus and Risk of Hepatic Fibrosis/Cirrhosis

**DOI:** 10.1155/2019/5308308

**Published:** 2019-04-04

**Authors:** Xu Li, Yuan Jiao, Yunlong Xing, Pujun Gao

**Affiliations:** ^1^Department of Hepatology, The First Hospital of Jilin University, Jilin University, No. 71 Xinmin Street, Changchun 130021, China; ^2^Department of Anesthesiology, China-Japan Union Hospital of Jilin University, Jilin University, No. 126 Xiantai Street, Changchun 130033, China; ^3^Department of Plastic Surgery, China-Japan Union Hospital of Jilin University, Jilin University, No. 126 Xiantai Street, Changchun 130033, China

## Abstract

Development of cirrhosis is two- to threefold greater in patients with diabetes mellitus (DM), and in this setting, the prevalence of cirrhosis is surging worldwide. The present review served to examine clinical ties between DM and liver fibrosis and hepatic cirrhosis and explore related biologic mechanisms. Pathways contributing to various etiologies of cirrhosis in conjunction with DM were key investigative targets.

## 1. Introduction

The estimated global prevalence of diabetes mellitus (DM), a metabolic disorder characterized by blood sugar and insulin dysregulation [1, 2], has an estimated global prevalence of approximately 9%, and by 2030, 300–400 million people will likely be affected worldwide [3], resulting in significant economic and social hardships [4, 5]. Unlike other chronic complications of DM, chronic liver disease (CLD) has been overlooked as yet another diabetic sequela, given the higher profiles of alternate pathogenic triggers. However, in many patients with cirrhosis, a major public health issue of global proportions, threatening the general population and imposing severe financial burdens [1, 2], the cause of which was once considered “cryptogenic,” DM is now accepted as a well-established cause [6]. Cirrhosis-related deaths are in fact increasing, totaling more than one million in 2010 alone [7]. Through a variety of mechanisms, cirrhosis clearly contributes to dysglycemia, whereas DM predisposes patients to serious liver disease [8].

At present, it is debatable whether type 2 DM is truly influential in the development and progression of liver disease if established risk factors for metabolic syndrome (i.e., obesity, hypertriglyceridemia) are lacking [9]. Furthermore, the risk of cirrhosis may be related to drug class or dosage of any particular antidiabetic agent prescribed [10, 11].

In this clinical review, we examine the association between changes in glucose metabolism and cirrhosis, the molecular mechanisms implicated in various etiologies of cirrhosis in patients with DM, and the relative risk of cirrhosis due to antidiabetic medications and DM duration.

## 2. Epidemiologic Studies Linking DM and Cirrhosis

### 2.1. DM and Hepatic Fibrosis

Cumulative evidence generated by prior research suggests that diabetes [12, 13], insulin resistance [13–18], and serum glucose [19] are associated with progression of hepatic fibrosis in patients with CLD. For example, in a 2001 multivariate analysis conducted by Ong et al. [20], advanced fibrosis was significantly more likely (odds ratio [OR]=6.5, 95% confidence interval [CI]: 1.1–38.5;* p* = 0.047) in patients with DM. Previous studies provided the following additional support: (1) a group of patients (N=201) infected with HCV genotype 1, studied by Petta et al. [13], establishing a significant link between concurrent DM and advanced fibrosis (OR=2.69, 95% CI: 1.46–4.95;* p* = 0.001); (2) significantly greater risk of advanced fibrosis (Ishak score >4) attributable to DM (OR=2.9, 95% CI: 1.2–7.1;* p* = 0.02) among HCV-infected patients (N=232) reported by Verma et al. [21], with comparable outcomes in instances of hepatitis B virus (HBV) infection; (3) a study conducted in Greece (1998–2003) confirming a relation between DM and more advanced fibrosis in HCV-infected patients (N=174) negative for hepatitis B e-antigen (HBeAg) (OR [model 1]=2.96, 95% CI: 0.95–9.22; OR [model 2]=3.87; 95% CI: 1.31–11.45) [22]; (4) a significantly higher incidence of DM in cirrhotic (vs noncirrhotic) subjects [23] determined by Huo et al. through a prospective study of Asian patients (N=500) chronically infected with HBV; and (5) retrospective analysis of 1365 patients with biopsy-proven nonalcoholic fatty liver disease (NAFLD), undertaken by Nakahara et al. [24], revealing that increases in prevalence of DM and degree of fibrosis paralleled one another. The latter is currently viewed as one of the largest exploits globally. DM was also deemed a significant risk factor for advanced fibrosis in such patients.

### 2.2. DM and Prognosis of Cirrhosis

Few studies have assessed the impact of type 2 DM on clinical outcomes of cirrhosis. In several cross-sectional retrospective analyses of patients with cirrhosis (any etiology), DM conferred greater risk of complications [25–27]; and the Verona study, enrolling >7000 individuals with type 2 DM, yielded a 5-year mortality risk 2.52 times (95% CI: 1.96–3.2) the at-large population risk [28]. When eligible subjects of two other investigations (retrospective: 354 patients, 98 diabetic; prospective: 382 patients) were monitored for a period of 6 years, only 110 survived. Remarkably, the higher DM-related mortality reflected greater risk of hepatocellular failure, as opposed to the impact made by more recognized diabetic complications [25]. Using varices as a covariate, subgroup analysis of 271 patients failed to identify DM as a risk factor, although its significance was restored by excluding mortality from gastrointestinal bleeding. In another study involving patients with cirrhosis and refractory ascites on the waiting list for liver transplantation, hepatocellular carcinoma (HCC) and DM emerged as independent predictors of mortality, whereas Child-Pugh score did not. Patients suffering from refractory ascites and DM showed 1- and 2-year survival rates of 32% and 18%, respectively. By contrast 1- and 2-year survival rates of patients with refractory ascites but without DM were 62% and 58%, respectively [29]. Nishida et al. performed OGTT on a group of 56 patients with cirrhosis and normal fasting blood glucose. Subsequently, 38% of patients were diagnosed with DM, 23% with glucose intolerance, and 39% were normal. Subsequent 5-year mortality rates in those with DM (44%) or glucose intolerance (32%) significantly surpassed the rate (5%) observed in those who tested normal, with only serum albumin and DM prevailing as independent negative predictors of survival via multiple regression analysis [30].

## 3. Biologic Mechanisms Linking DM and Cirrhosis

Mechanisms that worsen hepatic fibrosis or result in cirrhosis during the course of type 2 DM are complex and have not been clearly established. Firstly, DM promotes hepatic fibrosis and inflammation, exacerbating existing liver failure. Secondly, DM may facilitate bacterial infections in the context of cirrhosis, thereby increasing mortality [31, 32].

In relation to the first mechanism (i.e., hepatic fibrosis and inflammation), the disorders result from an increase in mitochondrial oxidative stress caused by excess triglycerides, resulting in free radical and peroxisome release [33, 34]. Adipokines (cytokines of adipocyte origin), such as leptin and tumor necrosis factor-*α* (TNF-*α*), are produced in excess [35]; and deficient adiponectin (a regulatory adipokine) enables an inflammatory adipokine milieu [36]. Ultimately, hepatic stellate cells (HSCs) are activated, boosting collagen production, connective tissue growth factor, and extracellular matrix, which then promote fibrosis and cirrhosis [37, 38].

Regarding the second mechanism (i.e., increased bacterial infection), DM may depress immune system function in cirrhotic patients, thereby increasing the incidence of severe infections, which may have deleterious effect on liver function. Hospital mortality rates are high in cirrhotic patients who spontaneously develop bacterial peritonitis, eventually succumbing to sepsis, liver failure, and hepatorenal syndrome. Additionally, patients with esophageal variceal bleeding have a high incidence of infections, therefore increasing their inpatient mortality rate [39]. However, it has not been determined whether DM increases the mortality rate in patients with other cirrhosis complications. Future studies should clarify the precise mechanisms by which DM may worsen liver function because treatment strategies targeting these mechanisms may reduce complications.

## 4. Relation between DM and Various Etiologies of Cirrhosis

Many reports have investigated the relationship between DM and various etiologies of cirrhosis. However, research has typically focused on DM, NAFLD, and HCV because of the complex interactions between them.

### 4.1. Hepatitis C Virus

HCV infection is still a major cause of liver fibrosis, HCC, and liver failure [40] though patients with chronic hepatitis C are decreasing recently due to the fact that a number of promising new direct-acting antiviral agents (DAAs) have been developed in the past few years [41]. DM is closely associated with HCV-related outcomes mentioned above.

Epidemiologic studies aimed at consequences of DM in the setting of HCV infection are limited, but available data suggests there is added risk for rapid progression of fibrosis, beyond that imposed by HCV alone [19, 42]. Such studies have focused on the chief liver-related outcomes of HCV infection (i.e., hepatic fibrosis, cirrhosis, and HCC) in examining the impact of DM/IR. Unfortunately, the studies undertaken are quite heterogeneous, and their outcomes often seem to conflict. In summarizing the published literature on HCV-infected patients and prevalence/risk of glucose abnormalities, Desbois et al. observed that glucose abnormalities and advanced liver fibrosis were related [43]. They reviewed 30 studies investigating a potential association between DM/IR and severity of hepatic fibrosis in HCV-infected patients, and in 26 of the 30 studies, this was indeed the case (OR range, 1.28–13.72) [43]. Hourigan et al. have also examined obesity and DM as predictors of liver fibrosis in patients with HCV infections, identifying a significant association between body mass index (BMI) and degree of steatosis on liver biopsy. Furthermore, they discovered a significant association between steatosis and hepatic fibrosis in a cohort with chronic HCV infections, implying synergism between steatosis and chronic viral hepatitis in the progression of hepatic fibrosis [44]. Similar findings also surfaced in a retrospective analysis of 286 consecutive HCV-infected patients conducted by Alsatie et al. Patients with DM were significantly more likely to display advanced fibrosis (Metavir stages 3-4) (OR=9.24, 95% CI: 2.56–33.36;* p*=0.0007) [45]. Huang et al. recorded an increased cumulative incidence of decompensated cirrhosis in conjunction with DM [46]. Finally, we have also previously demonstrated a 2-fold increase in the risk of cirrhosis for Chinese patients with chronic HCV infections and DM [47].

There have also been some seemingly negative studies in this regard [48–51]. When investigators used paired liver biopsies of HCV-infected patients to assess factors impacting progressive hepatic fibrosis over a specified period of time, DM was not among the independent predictors [22, 52].

The pathophysiologic mechanisms linking insulin resistance, diabetes, and chronic HCV infection have been intensively studied. Aside from those already mentioned, there are other possible explanations for cirrhotic risk under such circumstances. For instance, some researchers have found a relation between glucose and lack of sustained virologic response (SVR) to interferon alfa-based treatment [43, 49], corresponding precisely with the risk of cirrhosis. IR is a fundamental defect in type 2 DM and in the context of HCV may have bearing on failure to achieve SVR [16, 53–58]. In a prospective study of Spanish patients with chronic HCV infections [56], SVR was just 32.8% in those with IR harboring HCV genotype 1 (homeostasis model of assessment [HOMA-IR] >2), whereas in the absence IR (HOMA-IR ≤2), SVR was a more robust 60.5%.

### 4.2. Hepatitis B Virus (HBV)

HBV infection is an acknowledged global health problem, affecting approximately 250 million people worldwide [59]. Chronic infections may vary considerably in course, ranging from relative inactivity (with minimal viral replication or liver injury) to fulminant disease (with progressive fibrosis). At the latter extreme, there is the potential for the development of cirrhosis, liver failure, or HCC [60]. In addition to viral factors, certain host factors may also affect both disease course and long-term prognosis [61].

An abundance of evidence is available linking DM to risk of cirrhosis in HBV-infected patients, regardless of other major risk factors [23, 62, 63]. Results of a large population-based study conducted in Taiwan (1997–2009) have indicated that newly diagnosed DM in patients with chronic HBV-infections is independently predictive of cirrhosis, as shown by Cox proportional hazards model (HR=2.01, 95% CI: 1.39–2.91) adjusted for age, sex, HBV treatment, HCC, and comorbidity index [64]. Incidences of cirrhosis were 1.31 and 0.28 per 10,000 person-years in those with and without DM, respectively. In a similar Taiwanese study of chronically HBV-infected patients (N=516), DM emerged as an independent risk factor for cirrhosis in multivariate analysis, adjusting for age, sex, and persistent hepatitis (OR=5.2, 95% CI: 2.0–13.5) [62]. Still another effort undertaken in Greece (1998–2003), and enrolling 174 subjects, has determined that DM is associated with more severe fibrosis in patients with HBeAg-negative chronic HBV infections (OR [model 1]=2.96, 95% CI: 0.95–9.22; OR [model 2]=3.87, 95% CI: 1.31–11.45) [22]. Huo et al. addressed this issue further in a prospective study of Asian patients (N=500) with chronic HBV infections. In multivariate analysis, DM was significantly more frequent in cirrhotic than in noncirrhotic patients [23]; and in analysis by Mallet et al., DM was a proven risk factor for progression of liver disease, yielding an adjusted hazard ratio (HR) of 1.40 (95% CI: 1.32–1.48) [65].

In terms of related mechanisms, we know that patients with HBV DNA loads exceeding a certain threshold (≥10^5^ IU/ml) have fivefold greater risk of developing cirrhosis [66]. When exploring the relation between metabolic syndrome and HBV infection, Peter et al. encountered higher viral loads in conjunction with metabolic syndrome than in its absence [67]. The impact of DM on HBeAg seroclearance is the focus of an isolated publication [68], a Chinese study of chronically infected patients (N=413) who underwent liver biopsy or transient elastography between 2005 and 2012. Once adjusted for viral load, antiviral therapy, and necroinflammation, DM at baseline was confirmed as a predictor of delayed HBeAg seroclearance (HR=0.55, 95% CI: 0.32–0.97). Liver enzyme (ALT, AST, GGT) concentrations serve as nonspecific indices of hepatocellular damage (all causes). According to Peter et al., hepatitis B-positive patients with metabolic syndrome have shown higher ALT and GGT determinations, relative to patients without metabolic syndrome [67]. Wang et al. have also reported that BMI values >25 and hyperglycemia are independent predictors of ALT elevation, even at higher levels in normal range (upper half).

### 4.3. Nonalcoholic Fatty Liver Disease (NAFLD)

The spectrum of related disorders encompassed by NAFLD includes simple steatosis, steatohepatitis, hepatic fibrosis, and cirrhosis. Of these, the most benign is fatty liver, which, according to estimates, likely affects one-third of American adults [69]. Nonalcoholic steatohepatitis (NASH) represents the extreme manifestation of NAFLD, marked not only by steatosis, but also by tissue inflammation, cellular damage, and fibrosis. Although its prevalence is low, estimated at 2-3%, progression to cirrhosis and liver failure is presumed, and NASH is currently viewed as the commonest cause of cryptogenic cirrhosis [70, 71].

DM, metabolic syndrome, and concurrent NAFLD are likely culprits in progressive hepatic fibrosis and cirrhosis [72]. Whereas nonalcoholic CLD assumes greater importance in diabetic (vs nondiabetic) patients [73], Angulo et al. have found that obesity and DM predispose to development of NASH, constituting potential risk factors for more severe hepatic fibrosis, cirrhosis, and perhaps end-stage liver disease [74]. As mentioned earlier, Nakahara et al. [24] retrospectively analyzed the relation between metabolic factors and histologic severity of NAFLD in a large patient cohort (N=1365) with biopsy-proven NAFLD. Consequently, they found that prevalence of DM and degree of fibrosis showed parallel increases, identifying DM as a significant risk factor for advanced fibrosis in patients with NAFLD. Another study by Cazzy et al. likewise showed a significant association between type 2 DM and NAFLD in a morbidly obese population. This association entailed advanced forms of NAFLD, especially in the presence of NASH.

Other mechanisms may yet account for the above. High-level* in vitro* glucose and insulin concentrations common in patients with NAFLD are known to stimulate connective tissue growth factor expression, a pivotal event in progressive hepatic fibrosis [75]. In addition, a functionally deteriorating, cirrhotic liver may itself lead to the development of hyperinsulinemia and hyperglycemia [76, 77]. Hence, it is entirely feasible that DM both results from and perpetuates NAFLD [78].

### 4.4. Alcohol Abuse

Alcoholic cirrhosis is particularly common in Western countries. In a study conducted by Kikuchi et al., examining patients (N=1478) with alcoholic cirrhosis, DM was clearly implicated as a risk factor for cirrhosis [79]. Raff et al. also found that DM heightened the risk of cirrhosis and HCC in patients with alcoholic liver disease [80].

Alcoholic liver disease and NAFLD have similar pathogenic origins and histologic features, differing in phenotypes and risk factors [81] yet marked by a singular histologic continuum. Initially, there is simple steatosis, advancing to steatohepatitis and then hepatic fibrosis. The eventual endpoint is cirrhosis or HCC. As a risk factor for NAFLD, DM may worsen alcoholic liver disease [82], acting in synergy with alcohol intake [83, 84]. In addition, alcohol-induced oxidative stress may promote DNA damage and incite cirrhosis in diabetic patients.

## 5. Oral Antidiabetic Medications and Risk of Cirrhosis

*In vitro* and* in vivo* preclinical study results have shown evidence of hepatic damage or toxicity due to oral hypoglycemic agents. This poses challenges to DM management in patients with CLD, given that most antidiabetic agents (ADAs) are metabolized in the liver.

### 5.1. Metformin

As first-line therapy of type 2 DM, metformin is typically used to treat prediabetes and DM of lesser severity or more recent onset. Despite the noted benefits, namely, improved overall insulin sensitivity, reduced gluconeogenesis, and increased tissue utilization of glucose, its inherent mechanism of action remains unclear [85].

In rats with cirrhosis, metformin is known to reduce liver injury and improve hepatic fibrosis [10]. According to Doyle et al., metformin treatment reduced mortality and improved outcomes in HCV-infected and HCV/HIV coinfected patients with IR receiving direct-acting anti-HCV treatment [86]. Other randomized controlled trials (RCTs), however, have not corroborated this potential histologic transformation for NAFLD, whether in children or in adults [87, 88]. Currently, the molecular basis for antifibrotic effects of metformin is still subject to speculation.

High concentrations of metformin are achieved in the liver and appear to improve IR by activating AMP-activated protein kinase (AMPK), which reduces gluconeogenesis and increases uptake of glucose in skeletal muscle [89]. Results of* in vitro* studies indicate that this pharmacologic pathway may inhibit the transforming growth factor beta-1 (TGF-*β*1)-induced fibrogenic property of HSCs via transcriptional coactivator p300 [90]. Of the known fibrotic cytokines, TGF-*β*1 is best characterized [91]. Fan et al. have also shown that metformin treatment suppressed CCl4-induced elevation of liver enzymes, formation of fibrous septa, connective tissue accumulation, and upregulation of collagen I [10]. Activated HSCs are key in the progression of liver fibrosis and ensuing portal hypertension. In this regard, investigators have demonstrated the following actions of metformin: (1) inhibition of HSC activation; (2) inhibition of activated HSC proliferation, motility, and contraction; (3) reduced deposition of extracellular matrix; and (4) diminished HSC-induced angiogenesis. Thus, metformin treatment appears particularly advantageous in this setting [92].

### 5.2. Thiazolidinediones

Thiazolidinediones (TZDs) are peroxisome proliferator-activated receptor gamma (PPAR*γ*) agonists. Such agents reduce IR without altering insulin secretion directly [93]. In China, there are two TZDs (pioglitazone and rosiglitazone) currently available.

In a meta-analysis of eight RCTs evaluating TZDs, treatment for up to 24 months brought improvement in advanced or any-stage hepatic fibrosis, as well as resolution of NASH [11]. Use of pioglitazone seems especially beneficial for this purpose, exerting an inhibitory effect on hepatic inflammation and fibrosis in patients with NAFLD [94]. Even in the absence of DM, advanced fibrosis of NASH seemed to improve. Combined use of serelaxin and rosiglitazone for 2 weeks similarly has shown efficacy in significantly reducing established hepatic fibrosis, providing a potential new treatment strategy [95]. However, findings of another meta-analysis suggest that TZD therapy may reverse the histologic features of NASH while having no effect on advanced fibrosis [96].

### 5.3. Other Agents

Perhaps due to related hepatotoxicity, an experimental impasse was encountered regarding other antidiabetic drugs (i.e., sulfonylureas) and their impact on liver fibrosis. It is thus likely that any research attempts would meet ethical roadblocks.

## 6. Conclusion

DM is globally endemic, and there is increasing observational evidence linking it to cirrhosis. Consequently, increases in both are expected to climb. Use of metformin, a first-line agent for DM, seems to reduce the incidence of hepatic fibrosis/cirrhosis. It is unclear whether the above association is truly causal or merely reflects duration/severity of DM and whether the present body of data presented is skewed by bias or misclassification. In addition, the potential influences of antidiabetic drug class/dosage or duration of DM on the risk of cirrhosis must be further researched to better define the relation between DM and cirrhosis of various etiologies.
